# Identification of Leaf Promoters for Use in Transgenic Wheat

**DOI:** 10.3390/plants7020027

**Published:** 2018-03-28

**Authors:** Saqer S. Alotaibi, Caroline A. Sparks, Martin A. J. Parry, Andrew J. Simkin, Christine A. Raines

**Affiliations:** 1 School of Biological Sciences, Wivenhoe Park, University of Essex, Colchester CO4 3SQ, UK; saqer@tu.edu.sa; 2 Biotechnology Department, Biological Sciences College, Taif University, At Taif 26571, Saudi Arabia; 3 Rothamsted Research, West Common, Harpenden, Hertfordshire AL5 2JQ, UK; caroline.sparks@rothamsted.ac.uk (C.A.S.); m.parry@lancaster.ac.uk (M.A.J.P.); 4 Lancaster Environment Centre, Lancaster University, Lancaster LA1 4YQ, UK; 5 Genetics, Genomics and Breeding, NIAB EMR, New Road, East Malling ME19 6BJ, UK

**Keywords:** promoter, wheat, tissue specific, photosynthesis, reporter gene, yield

## Abstract

Wheat yields have plateaued in recent years and given the growing global population there is a pressing need to develop higher yielding varieties to meet future demand. Genetic manipulation of photosynthesis in elite wheat varieties offers the opportunity to significantly increase yields. However, the absence of a well-defined molecular tool-box of promoters to manipulate leaf processes in wheat hinders advancements in this area. Two promoters, one driving the expression of sedoheptulose-1,7-bisphosphatase (SBPase) and the other fructose-1,6-bisphosphate aldolase (FBPA) from *Brachypodium distachyon* were identified and cloned into a vector in front of the GUS reporter gene. Both promoters were shown to be functionally active in wheat in both transient assays and in stably transformed wheat plants. Analysis of the stable transformants of wheat (cv. Cadenza) showed that both promoters controlled *gus* expression throughout leaf development as well as in other green tissues. The availability of these promoters provides new tools for the expression of genes in transgenic wheat leaves and also paves the way for multigene manipulation of photosynthesis to improve yields.

## 1. Introduction

Over time, agricultural yields of our major crops, including wheat, have risen in line with demand due to improvements brought about through breeding and farming practices. World-wide, wheat is one of the most important agricultural crops providing in excess of 20% of the calories consumed on a daily basis [1]. However, yields of wheat have stagnated in recent years, while at the same time an estimated 70% increase in global supply will be needed by 2050 to meet the needs of the increasing global population [2,3,4,5]. Given that there is limited availability of land for cultivation, it will be necessary to develop higher yielding crop varieties in order to meet the predicted increases in demand [4,6]. This challenge is exacerbated further by the fact that improvements in yield will have to be realized with fewer resources and in a changing climate, where over the next forty years atmospheric CO_2_ levels are predicted to increase from the current level of 400 ppm to 550 ppm [7,8].

Recently, Driever et al. [9] generated transgenic wheat plants with increased yield through improvement of photosynthetic efficiency. These authors demonstrated that the over-expression of sedoheptulose-1,7-biphosphatase (SBPase) in wheat, an enzyme in the of the Calvin–Benson cycle, resulted in an increase in total biomass and dry seed yield (30–40% higher than wild type (WT)) in greenhouse-grown plants. This increase in yield was achieved either through a higher number of seeds being formed per ear (fewer tillers, at high plant density), or a larger number of ears being produced per plant (more tillers, at lower plant density) depending on the growing density [9]. Under field conditions, where environmental conditions can change rapidly, such a significant increase may be optimistic and manipulations involving multiple target genes of the Calvin–Benson cycle, photorespiration [10] and electron transport [11,12] may be required. Such multigene stacking approaches have been demonstrated in Arabidopsis [13] and tobacco [14] where a synergistic increase in biomass was observed. Due to the absence of a well-defined molecular tool-box in wheat, the simultaneous over-expression of multiple genes is more problematic.

Constitutive promoters are commonly used in plant studies to (over-)express genes of interest. Many of these promoters were identified in viruses such as the cauliflower mosaic virus (CaMV) 35S promoter [15,16] or the figwort mosaic virus (FMV) promoter [17,18]. Constitutive over-expression of transgenes can potentially result in gene silencing due to co-suppression [19,20,21] or alternatively have a negative impact on plant development, due to ectopic expression of the introduced gene. In response to this it was recognized that it would be important to identify and characterize promoters able to direct expression of the transgene in specific tissues. As early as 1989, the light-regulated and leaf-specific *Solanum tuberosum ST*-LS1 promoter was identified [22,23] as well as a number of Arabidopsis and tobacco photosynthetic tissue specific promoters [13,22], tomato fruit promoters [24,25,26], guard cell specific promoters [27,28,29] and promoters specific to seed endosperm [30,31,32,33,34]. Unlike many other species, for wheat there has been a dearth of promoters where the expression is restricted to green tissues. One reason for this is that it has proved to be difficult to use promoters from dicotyledonous species to drive transgene expression in wheat. 

Currently, the maize ubiquitin 1 [35] and the rice actin 1 constitutive promoters [36] are frequently used to direct expression of transgenes in wheat. Additionally, the semi-constitutive rice tungro virus promoter [37,38] has been used to express genes in the aerial parts of the wheat plant [9]. More recently, the rubisco small subunit (Rubisco) gene promoter from wheat was shown to direct expression in immature wheat embryos and tobacco leaves in transient assays [39]. Although this demonstrates the possibility that wheat promoters can be used to target transcripts to photosynthetic tissues, further work is needed to demonstrate that the wheat Rubisco promoter can function in leaves in stable transgenic wheat plants. A study using the rice and maize rubisco small subunit promoters in wheat to down-regulate expression of genes involved in photorespiration produced disappointing results [40]. Additional promoters will also be needed if we are to undertake multigene approaches. The reason for this is that it has been shown that the use of repetitious elements in transgenic constructs can have a negative impact on the stability and expression of the introduced genes [41]. The establishment of a robust tool-kit containing a number of well characterized promoters to use in future studies is therefore essential to facilitate multigene modification of traits in wheat. As a step towards the development of these genetic tools the aim of this paper is to provide information on new promoters for expression of genes in wheat leaves. We focused on the genes encoding the Calvin–Benson cycle enzymes, sedoheptulose 1,7-bisphosphatase (SBPase) and fructose 1,6-bisphosphate aldolase (FBPA), known to be expressed in green tissues [9,42,43]. Given that it has not proved possible to use promoter sequences from dicots in wheat and that using wheat gene sequences has also not always been successful in leaf tissue, we chose to identify gene sequences from the monocot grass *Brachypodium distachyon* (*B. distachyon*). The rationale underlying this choice is that *B. distachyon* is (1) a close relative of wheat and (2) that a fully annotated genome is available [44,45]. The results presented in this paper provide a clear demonstration that the promoters of the SBPase and FBPA genes from *B. distachyon* are functional in stably transformed transgenic wheat and direct expression in green leaf tissues. 

## 2. Materials and Methods

### 2.1. Construct Generation for Transient Expression Analysis in Nicotiana benthamiana Leaves

In order to isolate the DNA fragment of the SBPase promoter (Gene ID: Bradi2g55150) and FBPA promoter (Gene ID: Bradi4g24367) from the *B. distachyon* genome (see Phytozome website (https://phytozome.jgi.doe.gov/pz/portal.html)), specific primers (pSBPase fwd CACCTCGACGTCCATATGGCCCA; pSBPase rev TGCTGCGATGCGAGCTGC; pFBPA fwd CACCTCATTGGACGTGTTGATGTGC; pFBPA rev TGTTTCTGGCTCCAAAGG) designed from a region 2 kb upstream of the respective start codons were used (Supplementary Figures S4 and S5). The resulting 2 kb amplified products were cloned into pENTR/D as per manufacturer’s instructions (Invitrogen, Paisley, UK). The full-length promoter sequence was introduced into the pGWB3 gateway vector [46] (Supplementary Figure S1A AB289766) by recombination to make pGW:pSBPase::GUS (Supplementary Figure S1B) and pGW:pFBPA::GUS (Supplementary Figure S1C), respectively.

### 2.2. Construct Generation for Transient and Stable GUS Expression Analysis in Wheat

In order to analyze the expression of the SBPase and FBPaldolase promoters from *B. distachyon* in wheat tissue, the promoters were amplified using specifically designed primers (pSBPase fwd2 TTggcgcgccTCGACGTCCATATGGCCCA; pSBPase rev2 TTacgcgtTGCTGCGATGCGAGCTGC; pFBPA fwd2 TcccgggTCATTGGACGTGTTGATGTGC; pFBPA rev2 TTgaattcTGTTTCTGGCTCCAAAGG), with the addition of restriction enzyme sites (AscI and MluI sites in case of the isolation of SBPase promoter, and XmaI and EcoRI sites in the case of FBPA). The amplified sequences were cut with the relevant enzymes and then cloned separately into the pRRes14.041::GUS vector (supplied by Rothamsted Research, Harpenden, UK; Supplementary Figure S2A) at the equivalent sites to make pRRes:pSBPase::GUS Supplementary Figure S2B) and pRRes:pFBPA::GUS (Supplementary Figure S2C). 

### 2.3. Construct Generation for Stable Expression of a Small FLAG-Tagged Protein in Wheat Leaves

To evaluate the variation in transcript levels and the level of associated protein accumulation driven by these two promoters, a small 11 kDa protein (codon optimized *Porphyra umbilicalis* cytochrome *c*_6_ protein (AFC39870) [12]) with a FLAG tag (DYKDDDDK) was used. The pRRes:pSBPase::GUS and pRRes:pFBPA::GUS constructs (Supplementary Figure S2B,C) were cut with restriction enzymes NcoI and EcoRV removing the intron and the *gus* reporter sequence. The FLAG-tagged cytochrome *c*_6_ protein was cloned into the respective sites generating plasmids pSBPase::FLAG (Supplementary Figure S3A) and pFBPA::FLAG (Supplementary Figure S3B).

### 2.4. Agrobacterium-Mediated Transient Expression in Nicotiana benthamiana Leaves

Transient expression was carried out using 4- to 5-week-old *N. benthamiana* leaves [47]. *Agrobacterium tumefaciens* cultures (strain GV1301) carrying the promoter::GUS binary vectors or P19 plasmid were grown overnight at 28 °C in Luria Broth media with the appropriate selection criteria. Cultures were centrifuged at 4500× *g* for 15 min at room temperature and gently re-suspended in infiltration buffer (5 mM MES, 5 mM MgSO_4_, pH 5.7, 100 mM acetosyringone) to an optical density of OD_600_ = 0.6. Prior to infiltration, suspensions of *A. tumefaciens* carrying the binary plasmids which included the *gus* gene were mixed in a 1:1 ratio with the *A. tumefaciens* suspension harboring the viral silencing suppressor (P19) in the binary plasmid pBIN19-p19 [48]. The final mixtures of *A. tumefaciens* cells were infiltrated into the underside of two leaves from 4- to 5-week-old wild-type leaves from *N. benthamiana* plants. For controls, leaves were infiltrated with *A. tumefaciens* carrying the P19 binary plasmid only. Treated plants were housed in a 24 °C plant growth room using the 12 h/12 h light–dark cycle for three days [49,50].

### 2.5. Transient Expression in Wheat Leaves

Immature inflorescence sheath leaves which surround the young inflorescence were used as target tissue as these are metabolically active and are known to demonstrate good transient expression [51]. Young tillers were collected from ~6-week-old wheat plants (cv. Cadenza) cutting below the base node. A section ~10 cm long which contained the immature inflorescence (ranging in length 0.3–1.0 cm) was cut from each tiller and the ends were sealed with Parafilm^®^ (Sigma-Aldrich, Gillingham, UK). The trimmed stems were surface sterilized using 70% *v*/*v* ethanol for 3 min and 10% *v*/*v* domestic thin bleach (sodium hypochlorite content of 4–6%) for 3 min followed by several repeat washes with sterile, distilled water to remove the bleach. Sheath leaves were isolated by cutting away the outer leaf layers to expose the immature inflorescence and approximately 1–1.5 cm of the young sheath leaf closest to the inflorescence was removed. The isolated leaves were plated on L7 medium [52] supplemented with 3% *w*/*v* sucrose, 0.5 mg/L 2,4-D (Sigma-Aldrich, UK) and 10 mg/L AgNO_3_ (Sigma-Aldrich, UK), solidified with 5 g/L Agargel^TM^ (Sigma-Aldrich, UK) in 9 cm petri dishes, placing sufficient leaf sections to cover the central 2 cm^2^. The prepared leaves were transformed on the same day as isolation. Particle bombardment was carried out according to Sparks and Jones [53]. Essentially, each promoter::GUS construct was precipitated onto 0.6 µm gold particles (Bio-Rad, Perth, UK). The coated particles were delivered into immature leaves using the Bio-Rad PDS-1000/He^TM^ particle gun with a rupture pressure of 650 psi and a vacuum of 28–29′′ Hg. Bombarded tissues were cultured at 22 °C with a 12 h photoperiod. After 48 h, bombarded tissue was stained to assess GUS activity as described below.

### 2.6. Histochemical GUS Assays

Histochemical localization of GUS activity in situ was assayed according to Jefferson et al. [54]. Infiltrated and wild type *N. benthamiana* leaves or bombarded wheat tissues were immersed in GUS reaction buffer (1 mM X-Gluc (5-bromo-4-chloro-3-indolyl-β-d-glucuronide), 100 mM phosphate buffer pH 7.0, 0.1% *v*/*v* Triton X-100, 0.5 mM K_3_Fe(CN)_6_, 0.5 mM K_4_Fe(CN)_6_. The wheat leaves were vacuum infiltrated for a few minutes to promote penetration of the GUS reaction buffer. Tissues were then incubated at 37 °C overnight. Chlorophyll was removed from the leaves by soaking in several changes of 70% *v*/*v* ethanol until the blue GUS staining was visible. Tissue from wild type plants (WT) and plants transformed with the bar only construct (CN) were used as a control in this analysis.

### 2.7. Production and Selection of Transgenic Wheat Plants 

The recombinant plasmids pRRes:pSBPase::GUS, pRRes:FBPA::GUS (Supplementary Figure S2), pSBPase::FLAG and pFBPA::FLAG (Supplementary Figure S3) were introduced into wheat cv. Cadenza by particle bombardment of the scutellum of immature embryos, as described [53]. Essentially, each promoter::GUS construct was precipitated onto 0.6 µm gold particles (Bio-Rad, UK) alongside a selectable marker construct pRRes1.111 which contains the *bar* gene under control of the maize ubiquitin promoter with *nos* terminator to allow the selection of transformed material [35,55]. The coated particles were delivered into immature wheat scutella as described for transient expression analysis above. Bombarded tissues were cultured at 22 °C with a 12 h photoperiod following the subculture and selection regime as in Sparks and Jones [53]. A total of twenty-three independent transgenic lines were generated and confirmed to be carrying the pRRes:pSBPase::GUS construct and fifteen plants were confirmed to be carrying the pRRes:pFBPA::GUS construct using primers for *gus*: GusFwd: 5′ACTACGGGAAAGGACTGGAA′3 and GusRev: 5′GTCACAACCGAGATGTCCTC′3. The amplification reactions included 30 cycles of 1 min at 94 °C, 30 s at 57 °C and 45 s at 72 °C. Fifteen selected lines for construct pSBPase::FLAG (from a pool of 43 independent lines) and fifteen selected lines for construct pFBPA::FLAG (from a pool of 23 independent lines) were chosen and verified for the introduction of the cytochrome *c*_6_-FLAG tagged sequence using primers C6Fwd: 5′CCATGGCCTCCAACTCCCTCATGTCCTGC′3 and C6Rev: 5′CCAGCCCTTCTCGGACTGGGAGAGC′3. The presence of the selectable marker was verified using primers BarFwd: 5′GTCTGCACCATCGTCAACC′3′ and BarRev: 5′GAAGTCCAGCTGCCAGAAAC′3. The amplification protocol for the cytochrome *c*_6_-FLAG tagged sequence and selectable marker are as follows: reactions included 30 cycles of 1 min at 94 °C, 30 s at 57 °C and 30 s at 72 °C. These plants were self-pollinated and T1 and T2 plants used for further selection of stably transformed plants. Several lines were also retained as controls which had gone through transformation. These lines either contained no foreign DNA or had only the selectable marker *bar* construct present. Tissue was taken from transgenic lines, T1 and T2, at various stages of development to study *gus* expression; young leaves (~3 leaf stage), flag leaves, floral tissues and roots were assessed using a histochemical GUS assay as described above [54]. 

### 2.8. Plant Growth Conditions

Seeds of the T1 and T2 generations were germinated and seedlings were grown in compost (Levington F2S, Fisons, Ipswich, UK) in a climate controlled room for 3 weeks (22 °C, 12 h photoperiod). Selected seedlings were then transferred into 4 L pots (1 plant per pot), to a controlled environment greenhouse (25–32 °C day/18 °C night), with a 16 h photoperiod of natural irradiance (supplemented with high pressure sodium lamps, to a minimum light level of 600 μmol m^−2^ s^−1^). All plants were regularly watered and moved to minimize spatial variation of growth conditions [9].

### 2.9. RNA Isolation, cDNA Synthesis and qPCR

Leaf material (0.1 g fresh weight) was collected in 1.5 mL Eppendorf tubes and stored at −80 °C. Tissue was ground on dry ice in an Eppendorf tube and total RNA extracted from wheat leaf tissue as previously described [56] using the NucleoSpin^®^ RNA Plant Kit (Macherey-Nagel, Fisher Scientific, Loughborough, UK) and cDNA generated using the Fermentas RevertAid Reverse Transcriptase kit as per manufacturer’s instructions (Fermentas Life Sciences, Paisley, UK). Expression of the construct was determined from cDNA by qPCR and expressed relative to the gene expression of the wheat endogenous SBPAse [9]. Cytochrome *c*_6_ was amplified using primers Fwd: 5′ATCGACGCCATCACCTACCAGG′3 and Rev: 5′TCCTCGATGTCCTCGTCCACG′3 and the endogenous wheat SBPase with primers Fwd: 5′TCCAAGAACGAGATCATCCG′3 and Rev: 5′TTAGGCGGTGGCGCCGACGGTGG′3. qPCR reactions were performed using SensiFast SYBR No-ROX mix (Bioline Reagents Ltd., London, UK) as specified by the manufacturer. The amplification reactions included 45 cycles of 5 s at 94 °C, 10 s at 60 °C and 5 s at 72 °C and determined from nine technical reps. Fold expression was determined according to Pfaffl [57]. The PCR reaction of 25 µL total volume contained the equivalent of 100 ng of RNA transcribed into cDNA.

### 2.10. Protein Extraction and Western Blot Analysis for FLAG Protein

Leaf samples (0.2 g fresh weight) were ground in liquid nitrogen, extracted and quantified essentially as described by Harrison and Willingham [58,59]. Total protein (8 µg) of each sample was loaded onto a 12% *w*/*v* SDS-PAGE gel, separated and transferred onto a nitrocellulose membrane. The resulting membranes were probed using FLAG antibodies (Sigma-Aldrich, Gillingham, UK) and detected by ECL chemiluminescence detection reagent (Thermo Scientific, Rockford, IL, USA) using horseradish peroxidase conjugated secondary antibody and visualized by chemiluminescence (PEQLAB Ltd., FUSION FX chemiluminescence detection system, Sarisbury Green, Fareham, UK). 

## 3. Results

### 3.1. Identification and Analysis of Brachypodium distachyon SBPase and FBPA Promoters 

**Figure 1:**
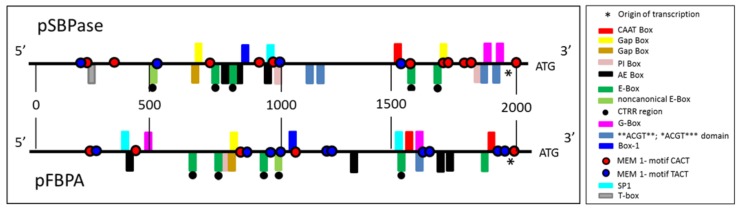
Schematic representation of a two-kilobase upstream region of the sedoheptulose-1,7-bisphosphatase (SBPase) and fructose-1,6-bisphosphate (FBPA) genes from *B. distachyon* showing potential regulatory motifs. ATG denotes the codon initiating translation of the SBPase and FBPA proteins.

To identify the promoter sequences from the *B. distachyon* SBPase (Bradi2g55150) and FBPA (Bradi4g24367) genes, the phytozome database (https://phytozome.jgi.doe.gov/pz/portal.html) was searched. A two-kilobase region upstream of the start codon (ATG) of both genes was isolated (Figure 1 and Supplementary Figures S4 and S5). The isolated promoter sequences were analyzed using PlantCare software (http://bioinformatics.psb.ugent.be/webtools/plantcare/html/) to identify and characterize the regulatory elements present in the promoters and these elements were compared with published material. Both promoters also contain a number of other cis-acting elements previously shown to be involved in light responsiveness (Figure 1 and Table 1).

### 3.2. Agrobacterium-Mediated Transient Expression Analysis in Nicotiana benthamiana Leaves 

**Figure 2:**
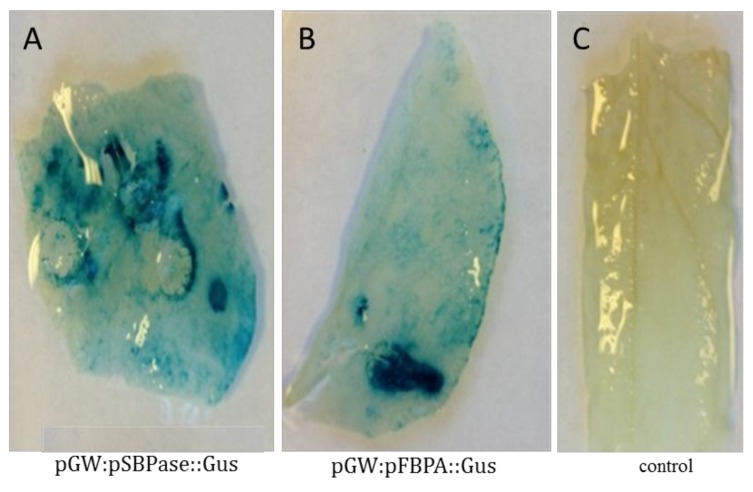
Histochemical GUS assay of agroinfiltrated *N. benthamiana* leaves. The transient expression assays were performed on four- to five-week old *N. benthamiana* leaves, incubated at 24 °C in 16 h/8 h light/dark for 72 h after infiltration prior to GUS staining: (**A**) *gus* expression driven by the SBPase promoter; (**B**) *gus* expression driven by the FBPA promoter; and (**C**) control (wild type: non-infiltrated leaf).

To test the functionality of the SBPase and FBPA promoters in our chimearic gene constructs, the *N. benthamiana* transient assay system was used. Our results showed that both of these promoters were able to drive expression of the β-glucuronidase gene (*gus*) in young leaves of *N. benthamiana* using the transcriptional fusion constructs pGW:pSBPase::GUS and pGW:pFBPA::GUS (Supporting Figure S1). Leaves of *Agrobacterium*-infiltrated plants were subjected to GUS staining and a clear, strong blue product was observed in the infiltrated leaves, which indicated *gus* expression driven by the SBPase promoter (Figure 2A) and FBPA promoter (Figure 2B). In comparison, no GUS coloration was observed in WT control leaves (Figure 2C).

### 3.3. Transient Expression Analysis in Wheat 

**Figure 3:**
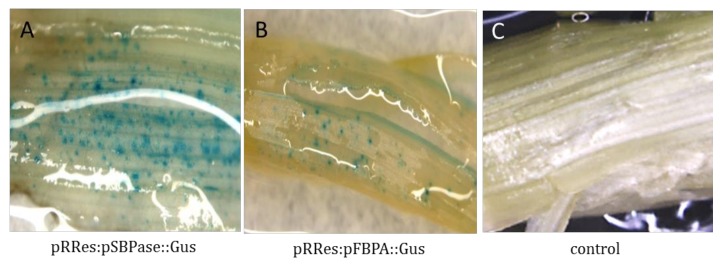
Histochemical GUS assay for transient expression of *B. distachyon* SBPase and FBPA promoters in wheat leaves. The transient assays were performed by particle bombardment of young wheat leaves which were incubated at 22 °C in 12 h/12 h light/dark for 48 h prior to GUS staining: (**A**) *gus* expression driven by the SBPase promoter; (**B**) *gus* expression driven by the FBPA promoter; (**C**) control leaf bombarded without DNA.

As a precursor to stable transformation in wheat, transient expression studies were carried out to test the functionality of both promoters to drive *gus* expression in wheat leaves using the constructs pRRes:pSBPase::GUS and pRRes:pFBPA::GUS (Supplementary Figure S2). Both promoters were shown to drive *gus* expression in wheat leaves. Many blue spots or patches were observed signifying that the *gus* transcript is being generated and translated into a functional protein (Figure 3). 

### 3.4. Stable Expression Analysis in Wheat

To study *gus* expression driven by the *B. distachyon* SBPase and FBPA promoters at different stages of wheat development and to confirm the suitability of these promoters for expressing transgenes in wheat, stably transformed lines were generated by particle bombardment of wheat scutella using constructs pRRes:pSBPase::GUS and pRRes:pFBPA::GUS.

**Figure 4:**
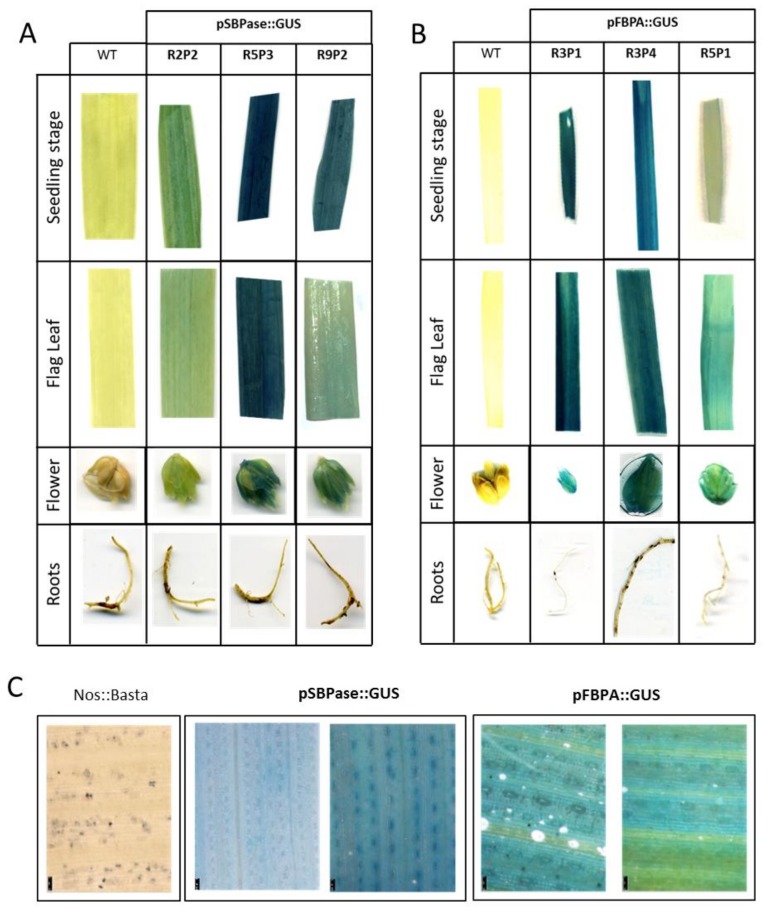
Histochemical analysis of GUS activity in T1 wheat lines stably transformed with the *B. distachyon* SBPase or FBPA promoter constructs. *gus* expression driven by (**A**) SBPase promoter and (**B**) FBPA promoter in tissue from young seedlings, from flag leaves of mature plants and in flowers and roots. WT = wild type infiltrated tissue is used as a control. (**C**) Microscopic observation of localization of *gus* expression in T1 wheat leaves of two independent lines for each construct compared to plants transformed with the *bar* (Nos:Basta) selectable marker gene construct only (pRRes1.111).

Samples were taken for GUS staining from a range of tissues from the T1 transgenic plants, including leaves (at two different growth stages: seedlings and flag leaves), roots and flowers. Three independent SBPase promoter transgenic lines (R2P2, R5P3 and R9P2; Figure 4A) and three independent FBPA promoter lines (R3P1, R3P4 and R5P1; Figure 4B), were analyzed and intense GUS staining detected in leaves and flowers, but, as would have been expected no expression was seen in the roots. GUS stained leaves of two positive lines expressing either the pRRes:SBPase::GUS or pRRes:FBPA::GUS gene constructs were subject to microscopic investigation. GUS staining was evident in both mesophyll and guard cells, but not in the midrib or veins of these plants (Figure 4C).

**Figure 5:**
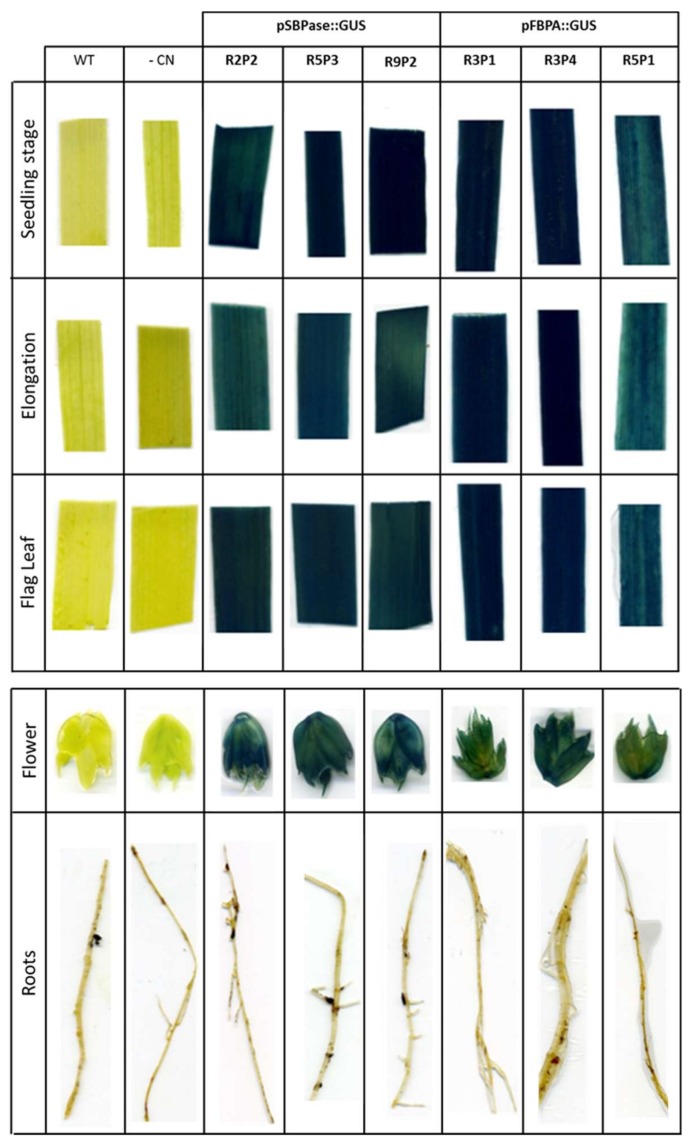
Histochemical analysis of GUS activity in T2 wheat lines stably transformed with the *B. distachyon* SBPase or FBPA promoter constructs. Leaves were taken for GUS staining at three different growth stages (seedling, elongation and flag leaf) together with samples from different tissues: flowers and roots. Three different lines per promoter were analyzed and compared to WT (wild type) and CN (control plants transformed with *bar* gene only).

To confirm the stability and suitability of the *B. distachyon* SBPase and FBPA promoters to drive the expression of *gus* in the next generation, GUS coloration was analyzed in T2 progeny arising from the lines studied above (Figure 5). Histochemical staining of leaves at three different growth stages (seedling, elongation and flag leaves), flowers and root tissues was carried out. GUS staining showed strong positive blue coloration in all three growth stages of leaves and in flowers of wheat plants, driven by either the *B. distachyon* SBPase promoter or FBPA promoter (Figure 5). Once again, no histochemical coloration of root tissue was observed with either promoter.

### 3.5. Evaluation of the SBPase or FBPA Promoters to Drive Expression of an Introduced Coding Sequence from Algae

**Figure 6:**
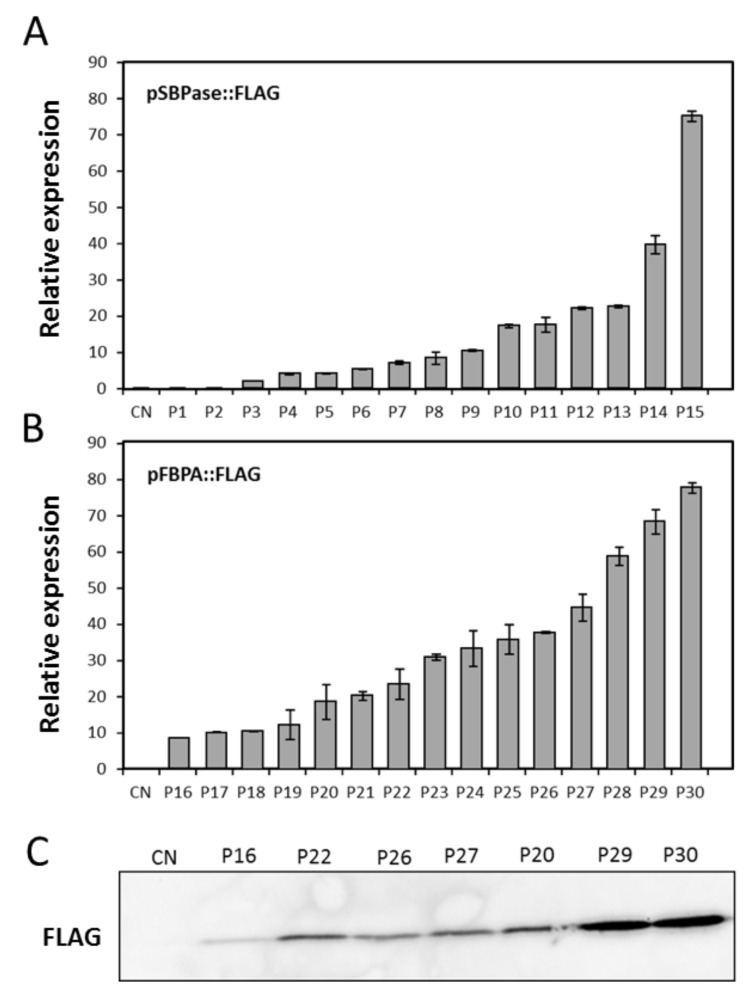
Analysis of transcript expression of the codon optimized *P. umbilicalis* cytochrome *c*_6_ [12]) with a FLAG tag (CytC6-FLAG) gene driven by the *B. distachyon* (**A**) SBPase or (**B**) FBPA promoters in stably transformed wheat T0 lines. Relative expression levels and range of expression in these 30 independent transgenic plants compared to the expression of the endogenous SBPase in the same plants. Data are presented as a % of the endogenous SBPase expression and standard error of nine technical reps are shown. (**C**) FLAG-tagged protein accumulation in a selection of FBPA promoter wheat lines.

To determine the range of expression levels that can be obtained using these promoters in stably transformed wheat, the SBPase or FBPA upstream sequences were fused to the coding sequence of a cytochrome *c*_6_-FLAG reporter (Supplementary Figure S3). The cytochrome *c*_6_-FLAG tagged construct was used to demonstrate successful expression of a potentially useful transgene other than *gus* in planta. Thirty confirmed primary transformed plants were analyzed by qPCR and a range of transcript levels obtained. Transcript levels were compared to the expression of the endogenous wheat SBPase gene. The SBPase promoter::cytochrome *c*_6_-FLAG transcript levels ranged from 0% to 76% of SBPase (Figure 6A) and the FBPA promoter::cytochrome *c*_6_-FLAG transcript levels ranged from 9% to 77% of SBPase (Figure 6B) in the first 30 transformed lines. Immunoblot analysis of seven selected FBPA promoter::CFLAG lines demonstrated that transcript levels also leads to an accumulation of protein (Figure 6C).

## 4. Discussion

In this study we have identified and tested two promoters from *B. distachyon* in wheat and shown that they can be used to drive the expression of the *gus* gene in mesophyll tissue and that expression is stable through subsequent generations. High levels of expression of introduced transgenes in wheat leaves have been difficult to achieve. Attempts to use dicot promoters, those from rice and even from wheat itself have often not been successful, with the promoters either failing or being ineffectual (author’s unpublished results). The work here has demonstrated that *B. distachyon* promoter sequences are functional in wheat, identifying a source of alternative promoters to those currently in use in wheat transformation, offering tissue-specificity to drive selected transgenes. We have also shown that *N. benthamiana* can be used to test promoters from *B. distachyon* prior to introduction into wheat. This is consistent with recent results which have shown that the rubisco small subunit gene promoter from wheat was functional in tobacco [39]. In plants expressing the FLAG-tagged cytochrome *c*_6_ protein under the control of either the SBPase or FBPA promoter, a range of transcript levels were seen between the individual independent primary transformants. This result is not unexpected and has been attributed to chromosome position, copy number or the presence of repeat sequences [60,61,62] and is known as “the position effect” [60,63,64,65]. The expression levels of this introduced gene can be as high as 80% of that of the endogenous SBPase transcript. The latter point is important as although GUS staining provides a convenient visual result for gene expression and tissue distribution, it did not provide information on the levels of expression relative to an endogenous gene. Although *gus* transcript levels could have been analyzed, it was important to demonstrate that these promoters could drive the expression of additional relevant transgenes and drive both transcript and protein accumulation. 

In this study we isolated a two-kilobase genomic region, directly upstream of the initiating ATG for the *B. distachyon* SBPase and FBPA genes, with the view that this region would contain both the core promoter elements (required for basal expression) and the cis-acting regulatory elements found in the extended region upstream of the core. Comparison of these two-kilobase genomic sequences to published literature allowed the identification of a number of DNA regulatory sequences. The SBPase and FBPA promoters were found to contain putative TATA and CAAT box sequences which, although not found in all plant genes, when present form part of the core region of the promoter. In the SBPase gene a motif (TCTATCTTCT) located 41 bp upstream of the transcription initiation site (indicated by the asterisk Figure 1) is a probable candidate for the TATA-box sequence [66]. In the FBPA promoter two candidate TATA-box sequences (ATTCATATCC; TCATATCCTT) are found 61 bp and 26 bp upstream of the transcription initiation site respectively [66]. Both the SBPase and FBPA promoters identified in this study contain a putative CAAT-Box [67]. The CAAT-Box contains the sequence 5′-CCAAT-3/5′-CAAAT-3 at its core and is a cis-acting regulatory element that potentially binds a number of different transcription factors. CAAT-Boxes are generally found upstream of the TATA box and although their position can be variable they are often reported to be 70–80 bp upstream (Table 1).

Directly relevant to this study it is interesting to note that both the SBPase and FBPA promoters also contain a number of YACT Mesophyll Expression Module (MEM) 1-motifs which have been shown to direct expression in mesophyll cells [68]. The function of the MEM motif was demonstrated in genes functioning in the C4 photosynthetic pathway which requires expression of genes to be separated spatially between bundle sheath and mesophyll cells. There is evidence that the gene sequences are also found in the C3 species but functional analysis in these plants remains to be confirmed [69]. 

There is very little information available on the regulation of expression of genes encoding enzymes of the Calvin–Benson cycle in wheat, with the exception of SBPase and FBPase [70,71]. However, there is a large literature demonstrating the role of light in regulating the expression of a number of Calvin–Benson cycle genes, across a range of species and this has led to the identification of a number light regulatory motifs. For example analysis of the GapB promoter (B subunit of chloroplast glyceraldehyde 3-phosphate dehydrogenase) from *Arabidopsis thaliana* in transgenic tobacco revealed that the ATGAA(G/A)A consensus sequence is necessary for light induced expression of the GapB gene [72]. The SBPase promoter contains two copies of these repeat sequences (ATGAAAA) and the FBPA promoter contains one similar motif (ATGAAGC). The GapA promoter (A subunit of chloroplast glyceraldehyde 3-phosphate dehydrogenase) contains 3 copies of a similar sequences CAAATGAA(G/A)A, denoted as the Gap Box; the deletion of just one of these copies results in a six-fold decrease in light induction and deletion of all three copies resulted in total loss of light induction [73]. Very similar sequences are also found in both the SBPase (AAAATGAAAA) and FBPA (CATATGAAGC) promoters isolated in this study. The Gap Box is not sufficient alone to confer light induction but it acts together with the Activation Element (AE) Box [74], with the consensus AGAATTCT sequence found in both the GapA and GapB promoters [75]. Here we have identified similar sequences in the SBPase (AGAACTCT; AGAATTCT; AGAAATAT) and FBPA (AGAAACAC; AGAAACAG; AGAATTGT; AGAATGTC) promoters (Table 1) indicating a similar regulatory mechanism might exist in monocot species. 

Both the SBPase and FBPA promoters were shown to contain a number of elements involved in directing light responsive gene expression (G-Box; CACGTG [76,77,78,79,80,81,82]; CACGTA). The G-box has been found in the promoters of circadian-regulated genes in plants [83,84] and is essential for phytochrome-regulated induction of transcription [77,85]. The related E-box core element (CANNTG), which has a significant overlap with the G-Box, is important in mammalian circadian promoters [86,87]. Interestingly in plants, the E-box CANNTG is involved in seed-specific expression of the French bean phaseolin [88], the 2S storage protein of Douglas-fir [89] and endosperm expression in coffee [34,90] was present in the *B. distachyon* SBPase promoter. A number of experiments have shown that the E-Box is an integral part of the circadian clock’s transcription-translation feedback loop [91,92]. Furthermore, a CT-region (CTRR) located approximately 23 bp upstream of the E-Box is considered to be essential for E-Box binding, transactivation and transcription of circadian genes in mice [91,92]. Each of the four E-Boxes identified here in the SBPase promoter have an identifiable CTRR domain 21, 9, 12 and 7 bp upstream of the E-Box. Corresponding E-box sequences were also identified in the FBPA promoter and were found to be in similar positions to those identified in the SBPase promoter. The first, second, third and fourth of these E-Boxes also followed identifiable CTRR domain at positions 13, 21, 14 and 14 bp upstream respectively [91]. Both the SBPase and FBPA promoter contain a single non-canonical E-Box previously shown to be essential for circadian expression in skeletal muscle [93] (Table 1).

Regulatory cis-acting elements previously shown to be involved in light responsiveness were also identified in both promoters (**ACGT** and *ACGT***). The motifs identified here are all similar to regulatory sequences previously identified in *Arabidopsis* [94]. These same sequences are also responsive to UV, drought and abscisic acid (ABA) and overlap with the G-Box motif CACGTC described in several plant promoters [65,66,67,68,69,70,71,72,73]. Other light responsive elements (Box-1 [95,96]) have also been identified in the SBPase and FBPA promoters as well as the light-responsive GAG-motif identified in the FBPA promoter [95]. Furthermore, sequences similar to the PI-box and T-Box identified in the GapB promoter [97] were present. The T-Box has also been identified in the geranylgeranyl pyrophosphate synthase promoter from *Ginkgo biloba* [98]. Mutations in the PI-Box and T-Box resulted in a reduction in light-activated gene transcription [97]. Furthermore, we also identified the SP1 motif in both the SBPase and FBPA promoters: SP1 is a light-responsive element previously identified in promoters from *Zea mays* [99] and *G. biloba* [98]. Although this analysis provides circumstantial evidence that the SBPase and FBPA promoters are regulated by light through a number of cis-acting motifs, further work is required to provide a functional demonstration that this aspect of the regulation is maintained in transgenic wheat. 

## 5. Conclusions

Here we present clear evidence that the genomic sequences from the promoter and associated upstream regulatory regions of both the *B. distachyon* SBPase and FBPA genes are capable of driving expression of transgenes in wheat leaves. Importantly, we show successful expression of the GUS reporter gene in multiple independent lines in both the T1 and T2 generations of transgenic wheat. Moreover, our data indicate that the levels of transcript expression attained when the algal cytochrome *c*_6_ gene is introduced into transgenic wheat, under the control of either the SBPase or FBPA promoter, can be close to that of the endogenous SBPase gene. Recent research has demonstrated that manipulating photosynthesis using multigene approaches can significantly increase biomass and seed yields in *Arabidopsis* and tobacco. The results presented here provide strong evidence that the *B. distachyon* SBPase and FBPA promoter sequences can be used as tools for the production of transgenic wheat, including multigene manipulation, with the goal of improving yield to meet global demand. 

## Figures and Tables

**Table 1:** Cis-acting elements analysis of 5′-upstream regulatory elements and their positions in the *B. distachyon* SBPase and FBPA promoters identified by PLANTCARE and literature search.

Name of the Element	Signal Sequence	pSBPase Position	pFBPA Position	Functional Description	Reference
**Activation Element (AE)-box**	AGAAAC(A/T)(A/T)	790, 859, 995	419, 1323, 1698, 1734	The AE Box and Gap Box act together and are essential components of light responsive elements	[74,75]
**ACGT-Box**	**ACGT** *ACGT***	1214, 1289, 1852, 1934	1584	Responsive to light, UV, drought and ABA	[88]
**Box-1**	TTTCAAA TTTGAAA	876	1163	Light responsive element	[89,90]
**CATT motif**	GCATTC CCAAT CAAAT	1554	1559 1892	Part of a light responsive element. Evolutionarily conserved in eukaryotic organisms, including fungi, plants, and mammals	[67]
**MEM1-motif** **YACT**	CACT TACT	241, 340, 755, 949, 1004, 1314, 1319, 1586, 1724, 1729, 1751, 1756, 2070 229, 593, 1082, 1569	205, 424, 857, 1176, 1260, 1988 226, 881, 982, 1046, 1243, 1250, 1600, 1666 1951, 1959	CACT key component of the Mesophyll Expression Module (MEM) 1 found in the cis-regulatory element in the phosphoenolpyruvate carboxylase promoter of the C4 dicot *Flaveria trinervia*	[68]
**E-Box**	CANNTG	769, 848, 1587, 1703	687, 775, 954, 1536, 1840	An integral part of the circadian clock’s transcription–translation feedback loop	[86,87]
**Non-canonical E-Box**	CAGCTT	515	1006	An integral part of the circadian clock’s transcription–translation feedback loop	[93]
**GAG-Motif**	AGAGAGT		146	Light responsive element identified in the rbcS promoter from Poplar	[95]
**Gap-Box**	ATGAA(G/A)A	724, 1709	830	Identified in the GapB promoter. Deletion of these repeats abolished light induction completely	[73]
**Gap-Box**	CAAATGAA(G/A)A	721	827	GapA promoter contains three sequences. Deletion of just one results in a six-fold decrease in light induction	[74]
**G-Box**	CACGTA CACGTG CACGTC GTACGTG TACGTG CACATGG CCACGTAA	1852, 1935	1585 598 1584	The G-box has been identified in the promoters of circadian-regulated genes in plants and is important for phytochrome-regulated transcriptional induction. Confers high-level constitutive expression in dicot and monocot plants	[76,77,78,79,80,81,82,83,84]
**TAAAGSTKST1**	TAAAG	941, 954	129	Target site for trans-acting StDof1 protein controlling guard cell-specific gene expression; KST1 gene encodes a K+ influx channel of guard cells	[100]
**PI-Box**	GTGATCAC GTGATCAG GTGATCAA TTGATCAC	1036	804 1847	Identified in the GapB promoter. Mutation resulted in a reduction in light-activated gene transcription	[97]
**T-Box**	ACTTTG	242		Identified in the GapB promoter. Mutation results in a reduction in light-activated gene transcription	[97,98]
**SP1**	CC(G/A)CCC	1013	1510	Light responsive element identified in *Zea mays*	[98,99]
**TGACG-motif**	TGACG	1288, 1742		Cis-acting regulatory element involved in the MeJA-responsiveness. Mutation of the motif in the 35S promoter causes a 50% drop in expression in tobacco leaves	[101]

## Supplementary Materials

The following are available online at http://www.mdpi.com/2223-7747/7/2/27/s1. Figure S1. Schematic of transcriptional gene fusion construct of the *B. distachyon* SBPase and FBPA promoters with the β-glucuronidase (*gus*) reporter for expression analysis in *N. benthamiana* leaves. Genomic DNA of *B.* *distachyon* leaves was used to amplify the native promoters of SBPase and FBPA. They were cloned into the pENTR vector (Invitrogen). The resulting product was transferred into the (a) pGWB3 vector by LR recombination to make (b) pGW:pSBPase::GUS and (c) pGW:pFBPA::GUS, Figure S2. Schematic of transcriptional gene fusion constructs of the *B. distachyon* SBPase and FBPA promoters with the β-glucuronidase (*gus*) reporter for expression analysis in wheat leaves. Genomic DNA of *B. distachyon* leaves was used to amplify the native promoters of SBPase and FBPA and they were cloned into the corresponding restriction sites (pSBPase was cloned into the Mlul and AscI restriction sites and pFBPA was cloned into the EcoRI and XmaI restriction sites) of the (a) pRRes14.041::GUS vector to make (b) pRRes:pSBPase::GUS and (c) pRRES:pFBPA::GUS, Figure S3. Schematic of transcriptional gene fusion constructs of the *B. distachyon* SBPase and FBPA promoters and coding sequences fused to the cytochrome *c*_6_-FLAG sequence for expression analysis in wheat leaves. The cytochrome *c*_6_-FLAG-tagged protein was cloned into the NcoI and EcoRV restriction sites of vector pRRes:pSBPase::GUS and pRRes:pFBPA::GUS, simultaneously removing the intron and *gus* reporter gene to generate (a) pSBPase::FLAG and (b) pFBPA::FLAG respectively, Figure S4. Sequence and regulatory motifs in the 2 kb upstream region of the *B. distachyon* SBPase gene. The different coloured boxed sequences represent the promoter motifs (see Figure 1), Figure S5. Sequence and regulatory motifs in the 2 kb upstream region of the *B. distachyon* FBPA gene. The different coloured boxed sequences represent the promoter motifs (see Figure 1).
