# Supplementary material for: Identification of Leaf Promoters for Use in Transgenic Wheat
Source: Plants (Basel). 2018 Mar 28;7(2):27. doi: 10.3390/plants7020027 (PMC6027260; doi:10.3390/plants7020027)
Supplement: Supplementary file 1 [file plants-07-00027-s001.pdf]

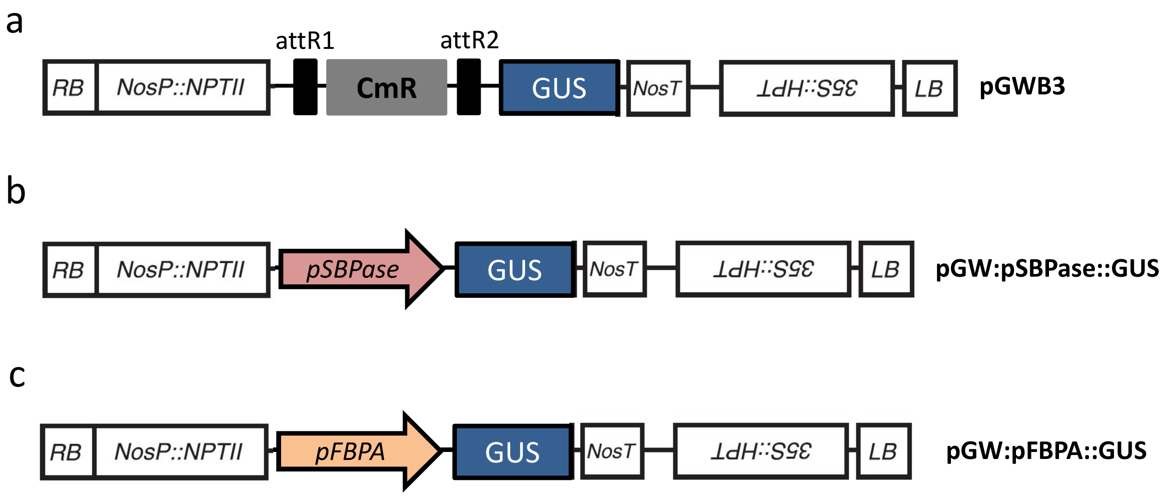

Supporting Figure S1. Schematic of transcriptional gene fusion constructs of the *Brachypodium* SBPase and FBPA promoters with the β-glucuronidase (GUS) reporter for expression analysis into *Nicotinia benthamiama* leaves. Genomic DNA of *Brachypodium* leaves was used to amplify the native promoters of *SBPase* and *FBPA.* They were cloned into the pENTR vector (Invitrogen). The resulting product was tranferred into the (a) pGWB3 vector by LR recombination to make (b) pGW:pSBPase::GUS and (c) pGW:pFBPA::GUS.

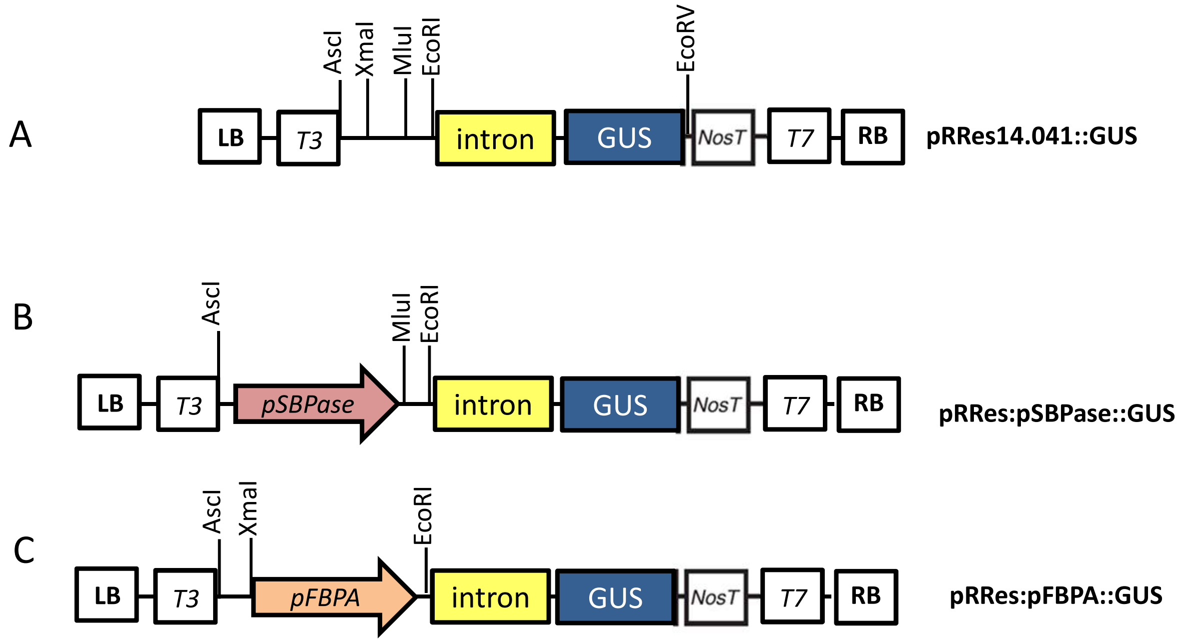

Supporting Figure S2. Schematic of transcriptional gene fusion constructs of the *Brachypodium* SBPase and FBPA promoters with the β-glucuronidase (GUS) reporter for expression analysis in wheat leaves. Genomic DNA of *Brachypodium* leaves was used to amplify the native promoters of SBPase and FBPA and they were cloned into the corresponding restriction sites (pSBPase was cloned into the MluI and AscI restriction sites and pFBPA was cloned into the EcoRI and XmaI restriction sites) of the (a) pRRes14.041 GUS vector to make (b) pRRes:pSBPase::GUS and (c) pRRES:pFBPA::GUS.

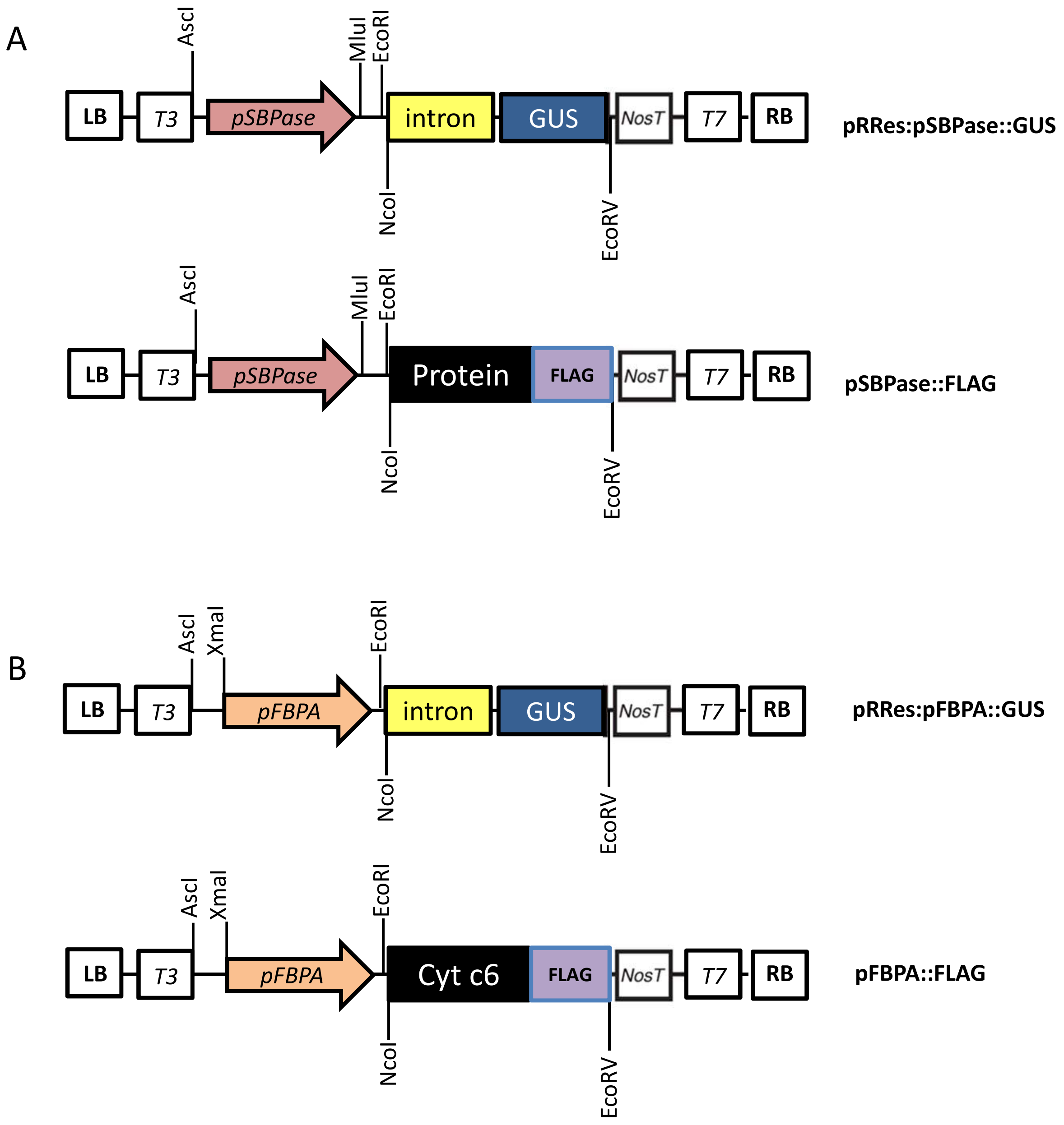

Supporting Figure S3. Schematic of transcriptional gene fusion construct of the *Brachypodium* SBPase and FBPA promoters and coding sequences fused to the cytochrome c_6-FLAG sequence for expression analysis in wheat leaves. The FLAG-tagged cytochrome c6-FLAG protein was cloned into the NcoI and EcoRV restriction sites of vector pRRES:pSBPase::GUS and pRRES:pFBPA::GUS, simultaneously removing the intron and GUS reporter to generate (a) pSneFLAG and (b) pFneFLAG respectively.

```
   1 TCGACGTCCA TATGGCCCAG GACGGCGGCT TCGCTCCTAG TATTACCTCT GTTCACATAT
  61 GAATTCTATG AATAGTAAGT TCTCCGTGTT GCGGTTGGTG ACAAGAATTT AGAATAGTAA
 121 TATTTTACGT GTGGTGTAAT TGACTGTCGC TGATTTTCTT AGACCATGAA ATCTTTCTTG
 181 GCCATGAAAA TGTGAAATCT TTCTTGACCA TGATTAGAGG TAAACTAGTA CTGTGTAGAA
 241 CACTTTGCCT TGGGAATTTT CAGATCCGTT AGTCTTAGGG AAATATGTTT TTTCCACCAA
 301 CCGGATTGTC TGCGAAGCTC TTTGTTTTGA TTCGTCTGTC ACTTGTAACT CAATAAGGTT
 361 GAGTTCTTTT CAGTTTCTAG GACCGGTTTT TTCTAAAAGC TGATCTCACC TAGCTTCTTA
 421 GAGAAGCCGC ATCTTAGATT TAGCTAGGTT TCCAAAATAA TTTGGTAGCC CAATCAAATT
                                          E Box
 481 TAGATGGTGA CTTCTTCAGG AAGCTGGGGG AGTGCAGCTT TTCGGAGAAG CCGATCCAGG
 541 AAACCGAAAA GAACTGGCCC TACGACGTCT ATTGATTTTC TCCGTCAGTT TTTACTTTTT
 601 AGAAACGTAG GTCTAGTTGA GCTATTGAAT AGATGGGCCT CTCATTTTGA TTTTGATTTG
 661 GGATTTGTCG AACCGAGTCA ACCTGTGGTG ATTTATAACC TCTCTAAATT TCCGATTCTC
        Gap Box                                                E Box
 721 AAAATGAAAA ACCTCTCTAA ATTTCCTAAA AAACCACTAT AAATAAGGCA AATGAGTGAA
                  AE Box
 781 AACAGGCTGA GAACTCTCGC TCCATATCCC GCCATATTTT TTCTCTTACA GCTCGTTTGA
           E Box          AE Box                Box 1
 841 CACCAATCAT CTGGGTTTAG AATTCTGAAA TTCATTTTCA AAAATCAACT TATTTGATTG
 901 GCGCAAGATT GGCCACGGAA ATTCAATCTC AAAAATTTCA TAAAGTCACA CTATAAAGTC
                                            AE Box             SP1
 961 CAATTCCAAA AATGGACGGT TAAATGGACG GCTAAGAAAT ATCCACTCCA TTCCACCCTG
                      PI Box
1021 TACGAAAGCA CAGTGGTGAT CAACAAGACA ACAAAGCTGG ATCAAATCCC TTGTCACGAA
1081 CTACTGCGGG CGGTGCCACG AAAAGCCAAC TTGCAAAGTA AGGTCCGGAT GAAACCTCCT
1141 AACTCCTAAG GCTAAGGGGC TGTTAAAACT TAATGTGGAT GCACCGTCTG ATGTAGATGA
                 ACGT Box
1201 ACTCCGAGGA ACAGACGTTG TGTCATTATT TCCGGACTCC AAGGGCGATT TTGTGGTGGC
                                    ACGT Box
1261 TACCTATAAG AAGTTTGCGG CGTGTCTTGA CGTTCATGTT CCGGAAGCTT CTGCACTACA
1321 CTTCGGACTC CTCGCACAAA TGGTGGGATG TAACAGGATG GTCATCGGTG GGTTGCACAA
1381 CCAGGAAATG ATTGGTACCG CGAGCCAGCA CGAGCGATGA GGTACGGTGG AGCTGCGATC
1441 TACGGAAACT CAAGTTTCAG GCAGACGAGT CCGTGGACAT TCGTGGACAT ATCCTTCCAA
                                                                CAAT Box
1501 CATGCGCACC GCGAGTCAAA CATGGTGGCA CACGAACTTG CTCCGATGGC AAAGCATTCT
                                  E Box
1561 CCCTCCGCTA CTTGGATCGA AACCCCACTT GGTAACATTA TACCTCTGCT CTTGGAAGAT
1621 GTAACGGCTA TTGAAGCCTG GTAAAATGGT ATTTGTGTCA AAAACAAAGA CAACAAAGCT
                               E Box  Gap Box
1681 CGATCTCTAC ACCTTAGGAA TTCAGTTGAT GAAAATAGCA GGACACTTCA CTGAATTTTT
1741 TTGACGAGTA CACTTCACTG AATTTAATCC GATGTGTTAC GACTTACAAG TCGCGACTAA
                                                   PI Box  G Box/ ACGT Box
1801 TTAATCTGCC CCTCCCTTGC AAGCGCGCAC ATACGTACGG ACCCGATTGA TCACGTCCCA
1861 GGAGGGCCAC ACAACCCACG GACGGTCGTC GCGCGCCCCG GGACGCTCCC GGCGCGCTCT
                G Box/ACGT Box                          *
1921 ATCTTCTCCC GCGCCACGTC GGCGCCCGGC TTATCTGGTC CAGTCGTGCC TCCGCCGTGT
1981 GTGATCCCAA ATCCCCCATC CGAGCTTAGC TGCAGCCGCA GGTAGGTCGC TCACCACGCA
2041 ACGTAAAATC GTATGACAAC TAAATAACAC ACTCCCCCCT CCAAAGAAAA GCTTAAGCTC
2101 AGTCCGCCTC GGTCACCTCG TCGGCGTCTA CCAGAGATTA CGGCGGCAGC TCGCATCGCA
2161 GCATG
```

Supporting Figure S4. Sequence of and regulatory motifs in the 2 Kb upstream region of the *Brachypodium* SBPase gene . The different coloured boxed sequences represent the promoter motifs (see Figure 1).

```
   1 TCATTGGACG TGTTGATGTG CTGCGAGAAG CTATCGCCAT TGCTGAGGCA GAAGCTTGTG
  61 GCTTTTTTAT CTAGTAGCAG TCCTGGAGCA GCTGAAGAAG CTTGTGGTAA ACAGATTAAT
 121 GAGGTTTCTA AAGCAGCAGA GTTGAAGAGA GTTTGCTCAA ACTAGATGTC ACAACCTTCT
 181 ATGTCCAGAA TGAACTACAG CCAGCACTAG TTTCCCAGCA ATTTGTACTT TTGTATGTTC
 241 CTGGAAAGGA AAGAATAAGC AGAGGAATCG CAAGGCTTTA CGAGTATCAC AATTCACAAG
 301 GAGTGGAGAT TGATGAACAC ATCTTTTGAA CAAGCAAGAG AAAGGAACTT GGCCCCTGCT
                                                 SP1
 361 TGCCTTCTGG AGCTACAAAC CTGGACCTCA CCAGCCACGG TCCACCCGGT ATGTATGGAG
       AE Box
 421 AAACACTCCC ATCAGAGGCT GCCCTGACAG TCAGATTAAA TCAGAAGTTA ACTTCATTTG
 481 TGTTCAACCA AATACATAAT GCCAGAGTAA ATGGAGATCA CGCATGATGC TTGATTTTCA
 541 ATTTCTGCTG GTTGAAACAA CTGAACTTAT GGTACCTTCG ATTGATATGC AGCCCAACAC
     G Box
 601 ATGGTATCTT TTGTTTGTTA CGGAGGATAG AATGCATTAA ACTGAAGGAT AGACTGTGCT
                                E Box
 661 TCCTGTGCTT CATTACAAGT TAGCTTCAGA TGTGATGAAT TTATGTGCTG GCAGCAAAGG
                                                                E Box
 721 AAATAATTGT CTGTGCAAAC CTATCTTCTG CCTGAACTGA TGAAAGAAAT GATACAGCTG
                              PI Box                       Gap Box
 781 CACAGTAGAT GCATTAAACG ATGGTGATCA GAGTTAAGGT CTGGTGCATA TGAAGCTTGT
 841 GATAGTGGAT GTTTTTCACT TTTTTGTTCA GGCATGCTCA TACTCTTTAC AGAAAAAGGC
                                                              E Box
 901 ACATACACCA CAGCAGGGAG TCTTGGATGT ATTTCTCCCG AGTTATAGGA GATCATCTGA
                                                       E Box
 961 TTTAAGTAGG CCAACCTTGT GTACTTGTGA ACAATTTGTA ACTATCAGCT TTTGTTATTC
1021 AATAGTAGTA AAATCCCTTT CATGGTACTC TGTGTCCTTT TTCATTGAGG GCAGTAGGTT
1081 CCATATACCA ACTTGCTCTG TGATGTTAAT GTTTTTGGTG ATTAGTGACA AGGGTGCCTA
                            Box 1
1141 ATTTCTTTTC TGATGTTAAT ATTTTGAAAG ATCTTCACTC TGAAAAAAAA ATGCATGTTC
1201 ATATCCATGT TTCTCTAAAA ATAATCCATA GCCAGTGTGT GATACTTTCT ACTAGTTCCC
1261 ACTAAATGCA TTGTGAAATT AAATTCTATA AAATTTTGTA ATTTCTAATA TTTAGTAAGG
         AE Box
1321 GTAGAAACAG AGATTTTTTT CTTAATTTTA AATCATCATG TATCAGGAGT CGCAAAGGTC
1381 CAGGAATGAT ATGCAAGATT GCTACATGGT TGGTATCCTC TTAATGTCAT CCTTGGCAGG
1441 GAGTTGTGGT GGATATGCCT TCTGCTGCCA GGCACTGGGG CACAAGAAGA ATGGTGTCTG
                SP1                         E Box
1501 CCACACAAGC CACCCTGCAC CCTACAAACA ACTCACAGCT GGAATGGTTA TCACCAAACC
    CAAT Box                  G Box/ ACGT Box
1561 AATGACAGAA AAAACTGTGT ATTCCACGTA ATTGATGGTT ACTGGCAAAA TTCATGGATG
1621 TAATACATCA GGGCATCTCA ACCGTCGAAA GATGCTCATG GGCACTACTC CTCGGCGAAA
                            AE Box
1681 TGCGCCCACA GAAACCCAGA ATTGTTCATC AGCCAAGACC ATCCTTAAGG TCAAGAATGT
                                                              AE Box
1741 CCAGATAATA TTTATGGACG GTGCAGCGCA AACGATAAAA TTCCAGTATT GCAGATTTTA
                                                 E Box
1801 CATGGTACAC AGAGAAGCTA AGGAAATCAT AGAGACAAGC ATGTGGCAGA GCCAGGACAA
                                      CAAT Box
1861 AAACAGAAGG TGGCAAGAGG ATTGGAGCAA CCAAATCACA GCCATTCATA TCCAGAAGGC
                                                               *
1921 CAGCCTCCAC CTCACAACTC ATATCCTTTG TACTCAGGTA CTCACCCTTA AATCTGAGCA
1981 GGCGCTTCAC TTCTCACCCC CCCTAAGGAA AGGCTGCAAT TGCAAGCTTG TGTCAAAGAA
2041 GAGGGTAGCA CCTGATCCTC TTGCCTTTGG AGCCAGAAAC AATG
```

Supporting Figure S5. Sequence of and regulatory motifs in the 2 Kb upstream region of the *Brachypodium* FBPA gene . The different coloured boxed sequences represent the promoter motifs (see Figure 1).

.
